# Brain sciences and the R words

**DOI:** 10.1093/braincomms/fcac283

**Published:** 2023-01-18

**Authors:** Graciela Muniz Terrera

**Affiliations:** Edinburgh, UK

## Abstract

Our Associate Editor, Graciela Muniz Terrera, discusses the importance of reproducibility in neuroscience and our special collection of papers on the topic.

Brain sciences are not foreign to the heated debate about reproducibility and replicability of scientific results that has occupied researchers in other fields. In addition to inadequate research practices and poor research integrity, several other reasons have been identified as culprits for replication and reproducibility failures.^1^ As a trained statistician, I am particularly concerned with inadequate analytical practices and their role in these failures. Easier access to data and code, rigorous code documentation and pre-registration of analyses are recommended open science practices that contribute to analytical transparency and enable peer-review scrutiny of analyses.^2^ Our community has started to embrace these recommendations and use resources such as data and code repositories developed with the open science framework to ease data access and code inspection, but not ubiquitously. Calls for change in institutions’ rewarding systems, funding and publishing culture have been made to accelerate their uptake,^2^ which I wholeheartedly echo.

Poor scientific communication and adherence to norms of scientific reporting have also been identified as contributors to replication or reproducibility failures.^3^ I find this especially troublesome and believe that it is an area that may require a mea culpa from the quantitative community. Guidelines for rigorous scientific reporting exist for many study types,^4^ but this is not yet the case for all study types and even when they exist, they are not consistently used.

As our community becomes more interdisciplinary, rigorous communication of all computational steps implemented becomes more necessary than ever before. The success of interdisciplinary teams depends on fluid dialogues between researchers with expertise in different areas, but these dialogues risk becoming monologues if we fail to communicate our work clearly to our collaborators and to the wider community. For these dialogues to be fruitful, efforts from all team members are necessary and the courage to get out of our research silos and expose our work and research practices to scrutiny are necessary.

I am hopeful that brain sciences researchers will accelerate the adoption of transparent research practices for the common benefit of our community and science.

Here at *Brain Communications*, one of our goals is to enhance rigour and reproducibility in translational neuroscience and we employ a scientific editorial team to check all our papers to be sure we have consistent reporting of statistical methods. In our special collection focusing on reproducibility in translational neuroscience, we highlight some of the papers in our journal that embody this ethos of rigour and reproducibility. These range from animal studies to human cohort studies to meta-analyses. The animal papers include coverage of conflicting results in a Pink1 knockout rat model of Parkinson’s Disease,^5,6^ replication of mouse model studies of immunotherapy for tau in Alzheimer’s disease,^7^ a study indicating that genetic background of mice is important for tau propagation,^8^ and negative data from a study in foetal sheep showing that erythropoietin does not augment hypothermic white matter protection after stroke.^9^ The human studies highlighted in the collection include a study exploring common genetic contributors to resilience to amyloid pathology,^10^ a biomarker study of senility with replications across cohorts,^11^ a call for harmonization of neuroimaging methods for studying Alzheimer’s disease,^12^ a registered protocol for a phase I clinical trail of fetal cell transplants in people with Huntington’s disease,^13^ a two centre blinded study of epilepsy patients undergoing surgery validating an algorithm on intracranial EEG to use high-frequency oscillations to guide epilepsy surgery,^14^ a population study in Korea of Graves’ disease and the risk of Parkinson’s disease,^15^ a study of subconcussive changes in youth football players replicating previous data observed in ice hockey players,^16^ and a study comparing subjective and objective cognitive decline.^17^ The collection also includes several systematic reviews and meta-analyses including a review of in vitro studies of toxicity of cerebrospinal fluid from people with amyotrophic lateral sclerosis,^18^ a meta-analysis of persistent neuropsychiatric symptoms after COVID-19,^19^ a meta-analysis of myelin-sensitive imaging for multiple sclerosis including recommendations for harmonized acquisition protocols,^20^ Together, the articles in this collection are excellent examples of how the translational neuroscience field can move forward towards more rigorous, reproducible data that eventually result in treatments that will work for all parts of the affected populations from diverse backgrounds.
